# 

**Published:** 1858-07

**Authors:** 


*The Cincinnati Lancet and Observer* is a new journal, formed by the fusion of the Cincinnati Medical Observer and the Western Lancet. The new journal is edited by Drs. Mendendall, Murphy and Stephens.

---

*Sounds of the Heart.*—In some recent papers published by Dr. Halford, in the London Medical Times and Gazette, some conclusive experiments are given, showing that the sounds are produced exclusively by the tension of the valves, the first by that of the auriculo-ventricular, the second by that of the semilunar valve.

*The Oglethorpe Medical and Surgical Journal.*—This is a new Medical Journal, issued by the Faculty of Oglethorpe Medical College, at Savannah, Georgia, and edited by two of the Professors, Drs. H. L. Byrd and Holmes Steele. It is handsomely gotten up, well supplied with original articles, and selected matter judiciously transferred. It is published every second month, the first number being issued in April.

---

*The New Jersey Medical and Surgical Reporter.*—We have received the following note from the Editor of this excellent journal, and cheerfully give it the publicity requested.—Eds.

Burlington, N. J., *April* 1, 1858.

From this date the Medical and Surgical Reporter will be issued from Philadelphia, and Wm. B. Atkinson, M. D., of that city, will be associated with the undersigned in the editorial management of the work.

The publishing department will be in the hands of Mr. J. W. Bradley, 48 North Fourth street, Philadelphia.

All communications, books, &c., should be directed to the Editors, care of Mr. Bradley.

☞ Exchanges will confer a favor by announcing the above change.

S. W. Butler, M. D.

*The American Dental Review.*—We have received from the Editor and Proprietor, Dr. A. M. Leslie of St. Louis, Mo. The first and second numbers of the above named periodical. We cannot say that the advent of this new candidate for professional honors and patronage, gives us pleasure, as we fear it will deprive us of one of our ablest and most highly prized correspondents, still we welcome the reception of it, most cordially, and wish it abundant success. After having borne this testimony to the ability of the Editor, it is unnecessary to speak of the manner in which it is conducted. It is published quarterly, each No. containing 48 pages.

*The Charleston Medical Journal and Review* has passed from the hands of Dr. Happoldt into those of Dr. J. Dickson Bruns, who is both editor and publisher.

*American Dental Convention.*—We have received from Dr. Taft, the Chairman of the Business Committee, the following communication, containing the subjects for discussion at the next meeting of the American Dental Convention. EDS.

SUBJECTS FOR DISCUSSION.

The Business Committee appointed at the convention at Boston, would most respectfully present the following topics for discussion at the next Convention to be held in Cincinnati in August next:

1. The best means of *securing* and *preserving* good teeth.
2. Treatment of exposed nerves.
3. Mechanical dentistry.
4. Filling teeth.
5. Miscellaneous.

J. TAFT,

C. W. SPALDING,

C. A. HARRIS,

E. TOWNSEND,

B. LORD,

} *Committee.*

